# Hypoxia-pretreated mesenchymal stem cell-derived exosomes-loaded low-temperature extrusion 3D-printed implants for neural regeneration after traumatic brain injury in canines

**DOI:** 10.3389/fbioe.2022.1025138

**Published:** 2022-09-30

**Authors:** Xiaoyin Liu, Jingjing Wang, Peng Wang, Lin Zhong, Shan Wang, Qingbo Feng, Xin Wei, Liangxue Zhou

**Affiliations:** ^1^ Department of Neurosurgery, West China Hospital, West China Medical School, Sichuan University, Chengdu, Sichuan, China; ^2^ Tianjin Key Laboratory of Neurotrauma Repair, Institute of Neurotrauma Repair, Characteristic Medical Center of People’s Armed Police Forces, Tianjin, China; ^3^ Department of Health Management, Tianjin Hospital, Tianjin, China; ^4^ The First Affiliated Hospital of Chengdu Medical College, Chengdu, Sichuan, China; ^5^ Department of Liver Surgery and Liver Implantation, State Key Laboratory of Biotherapy and Cancer Center, West China Hospital, Sichuan University, Chengdu, Sichuan, China; ^6^ Department of Urology, Institute of Urology, West China Hospital, West China Medical School, Sichuan University, Chengdu, Sichuan, China

**Keywords:** traumatic brain injury, exosomes, hypoxia, 3D printing, collagen, silk fibroin

## Abstract

Regenerating brain defects after traumatic brain injury (TBI) still remains a significant difficulty, which has motivated interest in 3D printing to design superior replacements for brain implantation. Collagen has been applied to deliver cells or certain neurotrophic factors for neuroregeneration. However, its fast degradation rate and poor mechanical strength prevent it from being an excellent implant material after TBI. In the present study, we prepared 3D-printed collagen/silk fibroin/hypoxia-pretreated human umbilical cord mesenchymal stem cells (HUCMSCs)-derived exosomes scaffolds (3D-CS-HMExos), which possessed favorable physical properties suitable biocompatibility and biodegradability and were attractive candidates for TBI treatment. Furthermore, inspired by exosomal alterations resulting from cells in different external microenvironments, exosomes were engineered through hypoxia stimulation of mesenchymal stem cells and were proposed as an alternative therapy for promoting neuroregeneration after TBI. We designed hypoxia-preconditioned (Hypo) exosomes derived from HUCMSCs (Hypo-MExos) and proposed them as a selective therapy to promote neuroregeneration after TBI. For the current study, 3D-CS-HMExos were prepared for implantation into the injured brains of beagle dogs. The addition of hypoxia-induced exosomes further exhibited better biocompatibility and neuroregeneration ability. Our results revealed that 3D-CS-HMExos could significantly promote neuroregeneration and angiogenesis due to the doping of hypoxia-induced exosomes. In addition, the 3D-CS-HMExos further inhibited nerve cell apoptosis and proinflammatory factor (TNF-α and IL-6) expression and promoted the expression of an anti-inflammatory factor (IL-10), ultimately enhancing the motor functional recovery of TBI. We proposed that the 3D-CS-loaded encapsulated hypoxia-induced exosomes allowed an adaptable environment for neuroregeneration, inhibition of inflammatory factors and promotion of motor function recovery in TBI beagle dogs. These beneficial effects implied that 3D-CS-HMExos implants could serve as a favorable strategy for defect cavity repair after TBI.

## Graphical Abstract

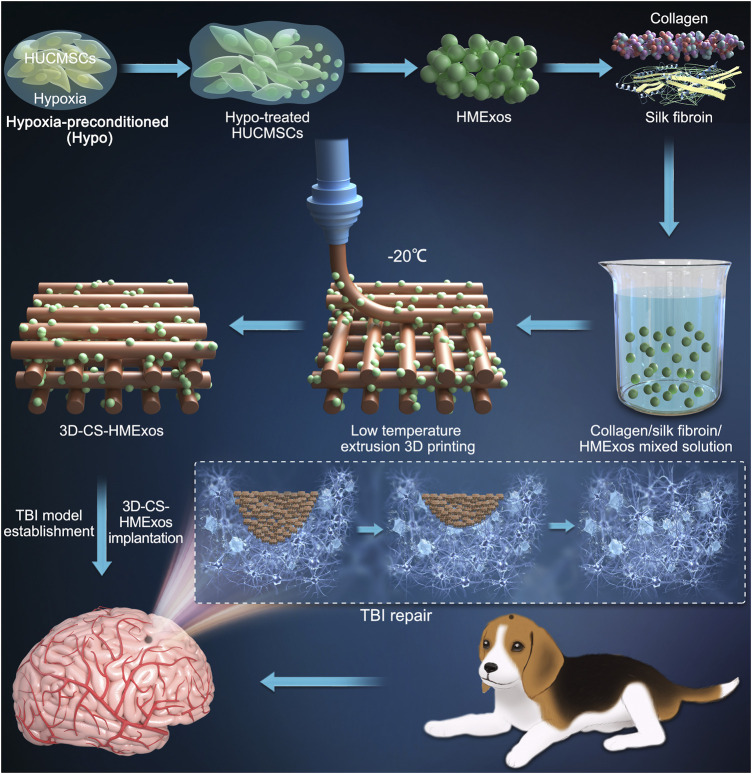

## Introduction

Currently, traumatic brain injury (TBI) is one of the key causes of unexpected death and disability in patients. The mortality rate of severe TBI is still as high as 30%, placing a heavy burden on society and families. The typical pathophysiology of TBI involves neuronal apoptosis, blood‒brain barrier (BBB) disruption, and neuroinflammation (Werner and Engelhard, 2007; Kinoshita, 2016); in particular, an ischemic and hypoxic microenvironment is formed *in situ* after traumatic brain injury, which aggravates neuronal apoptosis and hinders neuroregeneration. However, despite posing a serious threat to public health, TBI generally has no effective therapy.

As a subset of extracellular vesicles released from various cell types, we considered exosomes to be great delivery vehicles due to their intrinsic biological activity and ideal nanoscale size. Exosomes play an immunomodulatory role in repairing multiple cell damage caused by CNS lesions (Branscome et al., 2020) and have emerged as a novel therapeutic agent in regenerative medicine promoting the entry of cargo complexes into the cytoplasm. It has been reported that the treatment outcomes of mesenchymal stem cells (MSCs) are mostly due to their paracrine mechanisms, primarily involving the secretion of exosomes (Veneruso et al., 2019; Ha et al., 2020). The mechanism may be that the exosomes released by MSCs play a role in promoting repair, inhibiting inflammation, and regulating immunity by regulating the immune microenvironment (Shi et al., 2018; Liu H. et al., 2020).

Under different stress environments, the exosomes released by MSCs have differences in their functions and molecular regulation mechanisms, which in turn affect regeneration and repair. In previous studies, we observed that hypoxia preconditioning could enhance the proliferation and migration activity of MSCs and maintain MSCs in an undifferentiated state (Drela et al., 2014; Zhao et al., 2018). Studies have shown that hypoxia-induced exosomes derived from MSCs can stimulate efficient angiogenesis after spinal cord injury (Mu et al., 2022). More studies have also shown that MSCs cultured *in vitro* can release more exosomes after hypoxia, and these exosomes promote angiogenesis, improving myocardial blood perfusion and cardiac function of ischemic myocardium in infarcted rats (Bian et al., 2014). Hence, given the charater of exosomes in mediating injury repair and intercellular interactions, exosomes derived by hypoxic conditions may hold great promise in regeneration, such as angiogenesis in TBI.

Recently, a growing number of engineered scaffolds materials have emerged to deliver exogenous stem cells and have confirmed the repair efficacy of the defect cavity after TBI (Wang N. et al., 2018; Zhang et al., 2018). Biomaterials such as collagen, hyaluronic acid or silk fibroin can encapsulate stem cells inside the material and can be transplanted into the TBI defect. However, in the process of exogenous stem cell implantation, some outstanding problems still exist, such as ethics, oncogenicity, and immunological rejection, which hinder its translational application. According to recent progress in 3D printing technology and exosome repair, it is urgent to develop an engineered scaffold that can play a repairing role similar to that of stem cells. As a natural extracellular matrix constituent, prominent biocompatibility, low immunogenicity and suitable biodegradability of collagen have been observed (Wang et al., 2016; Sun et al., 2019). Studies have shown that collagen has been widely applied in various tissue engineering applications. However, the restriction of collagen in mechanical properties and degradation properties hinders its popularization and application. Unlike collagen, silk fibroin has become an alternative biomaterial for soft tissue reconstruction due to its good mechanical strength, elasticity, and environmental stability despite its shortcomings (Sun et al., 2015; Chen et al., 2022). Collagen complexed with silk fibroin can make up for the deficiency of using collagen scaffolds alone. The application of collagen combined with silk fibroin can make up for the deficiency of using collagen alone.

In summary, we surmised that combining collagen/silk fibroin scaffolds with hypoxia-preconditioned exosomes derived from MSCs (HMExos) could promote neuroregeneration and angiogenesis, reduce neuronal apoptosis and inhibit inflammation, thereby promoting TBI repair. In our study, 3D-printed collagen/silk fibroin/hypoxia-pretreated human umbilical cord mesenchymal stem cells (HUCMSCs)-derived exosomes scaffolds (3D-CS-HMExos) were prepared to carry and deliver exosomes. The appropriate porosity, water absorption and degradation properties of 3D-CS-HMExos heightened the adhesion and sustained release of exosomes, which were then transplanted to the site of injury. *In vivo*, after implantation to the injury site, the 3D-CS-HMExos implants were proven to possess good biocompatibility to enhance myelinization, axonal regrowth, and angiogenesis and ultimately enhanced motor functional recovery after TBI.

## Materials and methods

### Experimental animals and ethical statement

Male adult (1 year old) beagles, weighing 9–11.5 kg, were obtained from the Fang Yuanyuan Experimental Animal Center (Beijing, China) for this study. All animal experiments were carried out with the consent of the Fang Yuanyuan Experimental Animal Center (the license number: SK 2018-0026). All animal procedures were carried out according to the guidelines and were approved by the Institutional Animal Care and Use Committee of the Chinese People’s Armed Police Force (PAP) Medical Center and the institutional ethical and animal care committees. All experiments were approved by the Ethics Committee at the Institutional Animal Care and Use Committee of the Chinese People’s Armed Police Force (PAP) Medical Center [approval number is PJHEC-2019-02 (AF)].

### Establishment of a canine traumatic brain injury model and experimental grouping

The process of TBI model establishment was described previously (Jiang et al., 2018). Briefly, the beagles were anesthetized, and a bone hole approximately 3.5 cm long axis and 3 cm short axis over the right hemisphere was drilled at approximately 0.5 cm right of the midline using a hand-held cranial drill. We performed a standardized brain injury using a modified electronic cortical contusion impactor (eCCI, custom design fabrication, United States), in which the diameter of the impact probe was modified from 2 to 8 mm. Then, we set the parameters as 9.99 mm in depth, 5.34 m/s in speed and 255 ms in dwell time for the TBI model.

We randomly divided all 20 male beagles into six groups, including the Sham group (*n* = 5), TBI group (*n* = 5), 3D-CS-MExos group (*n* = 5), and 3D-CS-HMExos group (*n* = 5). Then, scaffolds with or without exosomes were implanted into the lesion immediately after the TBI model was prepared, followed by hemostasis and suturing. After the operation, the beagles were kept for recovery.

### Preparation of mesenchymal stem cells and neural stem cells

According to the method described previously (Dong et al., 2018), human umbilical cord mesenchymal stem cells (HUCMSCs) were isolated from Wharton’s jelly from the umbilical cord provided by the Department of Obstetrics and Gynecology of the Characteristic Medical Center of People’s Armed Police Forces, and informed consent was obtained. After removing the umbilical artery and umbilical vein and their remaining blood, we cut the umbilical cord Wharton’s glue into pieces approximately 1–2 mm^3^ in size and then digested them with a mixed solution of 0.2% hyaluronidase and 0.2% collagenase II to extract the primary HUCMSCs. The p3-generation HUCMSCs were used for the following experiments. The morphology of HUCMSCs was captured by using a phase-contrast microscope.

Primary neural stem cells (NSCs) were isolated from embryonic (E) day 14 Sprague−Dawley rats as described in a previous study (Dai et al., 2019). In brief, the hippocampal tissue was dissociated into a cell suspension and seeded into complete growth medium containing DMEM/F12 culture medium, 20 ng/ml EGF, 20 ng/ml bFGF, 1% N2, 2% B27, and 4 mM glutamine at a density of 1 × 10^6^/ml. After 5 days, the NSCs grew into neurospheres and were then digested mechanically for passaging. The third-generation neurospheres were collected for the subsequent experiments. For NSC identification, the NSC neurospheres were identified with an NSC-specific marker nestin antibody by immunofluorescence staining staining and observed by using a fluorescence microscope (Leica DMI4000B, Germany).

### Hypoxic preconditioning treatment of mesenchymal stem cells and collection of exosomes

HUCMSCs cultured above were seeded, and FBS was depleted of exosomes by ultracentrifugation at 100,000 × g for 18 h using an XPN-100 ultracentrifuge (Beckman Coulter, United States) at 4°C. HUCMSCs were plated in T75 cell culture flasks, and hypoxia treatment was conducted after cell adhesion. HUCMSCs were incubated under normoxic conditions (21% O_2_, 5% CO_2_) and hypoxic conditions (1% O_2_, 5% CO_2_) at 37°C for a total of 24 h. After HUCMSCs reached 80% confluence, the culture supernatant was harvested for exosome isolation.

### Isolation and characterization of MExos and hypo-MExos derived from human umbilical cord mesenchymal stem cells

For exosome isolation, normoxic and hypoxic exosome-containing supernatants were separated and purified by differential centrifugation. The supernatant was prepared by centrifuging at 300 × g for 10 min to remove the cell pellet; subsequently, at 2000 × g for 10 min to further remove the cell pellet; then, the sample was passed through a 0.22 μm filter (Millipore, SLGP033RB); and finally, at 10,000 × g for approximately 1 h, the precipitate was collected, which contained the crudely purified exosomes. The exosomes used in this study were further purified by centrifugation at 100,000 × g at 4°C for 18 h using a Himac CP100NX centrifuge (Hitachi, Japan). Finally, we resuspended the exosome particles in PBS. The collected exosomes were deposited at −80°C for the subsequent experiments.

We characterized the collected exosomes by using a specific surface marker, covering CD9 antibody (Proteintech, China), and CD63 antibody (Wuhan Servicebio Technology Co., Ltd., China). TEM (Tecnai G2 spirit, Thermo FEI) and nanoparticle tracking analysis (ZetaView x30, DEU) were applied to observe the morphology and size distribution of exosomes, respectively. The exosome concentration was determined and quantified by a bicinchoninic acid (BCA) protein assay kit (Beyotime, China).

### Fabrication of the exosomes loaded 3D-printed collagen/silk fibroin scaffolds

A 3D-Bioplotter™ system (Regenovo, Hangzhou, China), including a personal computer, x–y–z motion nozzle and temperature controllers platform, was served for printing scaffolds. For biocompatibility and biodegradability, a blend of collagen and silk fibroin was prepared as the fabrication material as described previously (Jiang et al., 2021; Li et al., 2021; Chen et al., 2022). For collagen (Wang Y. et al., 2018; Liu et al., 2019; Jiang et al., 2020), we purchased fresh bovine tendons from a local slaughter house. After isolating aponeurosises with a thickness of 0.5 mm, we further excised the aponeurotic attachments, including connective and adipose tissue. Then, we soaked the samples in 0.05 Mtris buffer for 24 h for further purification. The supernatant was prepared by adding pepsin-containing acetic acid to the pellet. Subsequently, 3.5 mol/L NaCl was added to the supernatant for salting. Finally, purified collagen was obtained by centrifugation of the salting precipitate and dialyzed against deionized water for 5 days. Silk fibroin (Ruan et al., 2011; Xu et al., 2016) was fabricated with silkworm silk, incubated in 0.5% Na_2_CO_3_ solution at 100°C for 30 min and dried. Then, the CaCl_2_ CH_3_CH_2_OH H_2_O solution was affiliated at 70°C and stirred until dissolved. Then, a certain concentration fibrin solution is arrested after the steps of dialysis, filtration and concentration.

In the current study, collagen was blended at 1:6, 1:9, 1:12, 1:15, and 1:18 ratios with silk fibroin to investigate the optimal molding parameters of 3D printing technology. Finally, we chose a ratio of 1:12, which is sufficient to achieve the best repair effect. To encapsulate exosomes in the 3D-printed scaffolds, 0.1 g collagen/chitosan mixed solution was mixed with 200 μg of MExos or Hypo-MExos resuspended in PBS individually. The 3D-printed scaffolds were formed at a low temperature of −20°C with the following printing parameters: nozzle diameter = 160 μm, extrusion speed = 0.17 mm/min, printing speed = 12 mm/s, and thickness = 0.3 mm per layer. There were five main types of scaffolds fabricated in our study: collagen/silk fibroin scaffolds (CS), Hypo-MExos-loaded collagen/silk fibroin scaffolds (CS-HMExos), 3D-printed collagen/silk fibroin scaffolds (3D-CS), MExos-loaded 3D-printed collagen/silk fibroin scaffolds (3D-CS-MExos), and Hypo-MExos-loaded 3D-printed collagen/silk fibroin scaffolds (3D-CS-HMExos). Next, 3D-CS scaffolds were placed at −80°C overnight and freeze-dried for 48 h. Finally, after repeatedly rinsing in deionized water, we divided the 3D-printed scaffolds into cylindrical scaffolds of 2 mm in diameter and 2 mm in height and sterilized them with Co^60^ radiation. To prove the distribution of exosomes in 3D-CS-HMExos, exosomes microscopy and adhesion were observed with scanning electron microscopy (SEM, Hitachi, Tokyo, Japan) and confocal laser scanning microscopy (CLSM, LSM 880, Zeiss). 3D-CS-MExos and 3D-CS-HMExos were incubated with D-Hank’s solution for 30 min. We implanted 3D-CS-MExos and 3D-CS-HMExos into the canine lesion site after incubation.

### Scanning electron microscopy and TEM assay

3D-CS-HMExos were investigated with SEM. Briefly, we prepared 3D-CS scaffolds with or without exosomes. The crosslinked construct was fixed with 70% ethanol for 90 min and then 100% ethanol for 30 min. Immediately thereafter, the fixed samples were freeze-dried in a freeze dryer for 2 days and then coated with platinum for SEM imaging. For the TEM assay, exosomes were prepared as described above. After rinsing the mesh copper grids, the samples were placed on the grids and incubated for 30 min. The excess-free sample droplets were absorbed into a tissue to ride off the remaining particles. Finally, the samples were stained with 2% uranyl acetate for 10 min. The morphology of exosomes was observed by TEM.

### Evaluation of physical properties

Scaffolds were prepared as mentioned above and placed in phosphate buffered saline (PBS, pH 7.4) at 37°C. *In vivo* degradation experiments of scaffolds at 1, 2, 3, 4, 5, and 6 months after scaffolds implantation were performed according to previously published study (Jiang et al., 2020). The water absorption was determined by examining the wet weight of each scaffold after a certain time (Zeng et al., 2015). The estimation formula of the water absorption rate is Ws/Wo × 100%, where Wo and Ws represent the wet weight of the initial stent on day 0 and the wet weight of the swollen stent at a certain time point, respectively. The porosity of the scaffolds was obtained according to the established formula: Porosity ratio (%) = (V1 − V3)/(V2 − V3) × 100%. A BCA protein assay kit (Beyotime, China) was performed to evaluate cumulative release profile of exosomes from 3D-CS-HMExos and CS-HMExos at 2, 4, 6, 8, 10, 12, and 14 days according to the published literature (Guan et al., 2022).

### Fluorescence staining and cellular uptake of exosomes by human umbilical vein endothelial cells and neural stem cells

The red fluorescent dye PKH 26 (Sigma‒Aldrich, United States) was used for exosome staining according to the instructions. The remaining dye solution was subjected to repeated ultracentrifugation at 100,000 × g for 70 min at 4°C. For cellular uptake, HUVECs and MSCs were incubated with labelled exosome suspension (30 μg/ml) for 4 h at 37°C. Subsequently, F-actin was stained with phalloidin (Beyotime, China) before being observed.

### Cell proliferation and adhesion evaluation

To carry out the cell proliferation test, 1 × 10^6^ HUCMSCs or NSCs were cocultured with 3D-CS-MExos and 3D-CS-HMExos for 1, 3, 5, and 7 days. The culture medium was replaced with CCK-8 solution (Solarbio) and incubated for 3 h. Then, the absorbance of the supernatant was measured at 450 nm with a microplate analyser (BioTech). The adhesion of NSCs to the scaffold was tested by the following method. Briefly, NSCs at a density of 1 × 105 per well were cocultured with 3D-CS-MExos and 3D-CS-HMExos for 3 days, fixed with 4% paraformaldehyde, and stained with F-actin and DAPI, and the morphology of the NSCs was detected by CLSM.

### Neurological examination

As described in our previous study (Liu et al., 2022), a modified Galasne score system (mGCS) was prepared and evaluated by two individuals blinded to the experimental groups at 1, 2, 4, 8, 12, 16, 20, and 24 weeks after surgery. The mGCS score scale (Platt et al., 2001) (total score range from 3 to 18) evaluates the recovery of brain neurological function according to the level of scores, of which 3 scores indicate brain damage and 18 scores represent healthy brain. Meanwhile, NDS scores (Castellá et al., 2005) and Purdy scores (Wu and Zhong, 2010) were calculated at the same time point as the above mGCS score to further verify the recovery of neurological function. On the NDS scale, 0 scores represent healthy brains, and 500 scores suggest brain damage, while on the Purdy scale, a score of 2 indicates healthy brains, and a score of 11 suggests coma or death (*n* = 5 for each group).

### Immunofluorescence staining and TUNEL assay

After cardiac perfusion, the beagles were sacrificed. The harvested brains encompassing the lesion site were fixed in 4% paraformaldehyde (PFA) and then prepared for frozen sections. Specific surface markers for neurogenesis (MAP-2: microtubule-associated protein 2, Tuj-1: neuronal class III β-Tubulin and NF: neurofilament protein), angiogenesis (vWF: von Willebrand factor), myelination (MBP: myelin basic protein), axon membrane protein formation (GAP43: growth-associated protein-43), and synapse formation (PSD95: postsynaptic dense protein 95 and Syn: synapsin) were detected. Primary antibodies against NF (Proteintech, China), MBP (Abcam, United Kingdom), GAP43(Proteintech, China), Tuj-1 (Abcam, United Kingdom), Syn (Bioss, China), MAP-2 (Abcam, United Kingdom), PSD95 (Proteintech, China), and vWF (Abcam, United Kingdom) were used and incubated with the slices overnight at 4°C. Subsequently, secondary antibodies (Life Technologies, United States) were incubated at 37°C for 1 h. Nuclei were stained with DAPI (Sigma, United States). Finally, the sections were observed under a fluorescence microscope (DMI4000B, Leica, Germany).

TUNEL staining (Promega Corporation) was used to detect the apoptotic cells in the lesion in each group at 6 months as described previously (Zhang J. et al., 2021). The numbers of TUNEL-positive neurons were recorded in five fields that were randomly selected under the microscope at ×200 magnification. The ratio of apoptotic neurons was calculated by the following equation: TUNEL-positive neurons/total neurons) ×100%.

### Measurement of plasma inflammatory factors

After the serum of each group was harvested at 1 week and 6 months, the levels of IL-10, IL-6, and TNF-a were detected by an ELISA kit (Wuhan Servicebio Technology Co., Ltd., China).

### Statistical analysis

All data are expressed as the mean ± SD. Statistical analyses were processed by SPSS 22.0. Statistical differences between groups were compared by one-way ANOVA. Statistical significance was defined as **p < 0.01, *p < 0.05, ^##^ *p* < 0.01, and ^#^ *p* < 0.05.

## Results

### Preparation and characterization of MExos and Hypo-MExos

**FIGURE 1:**
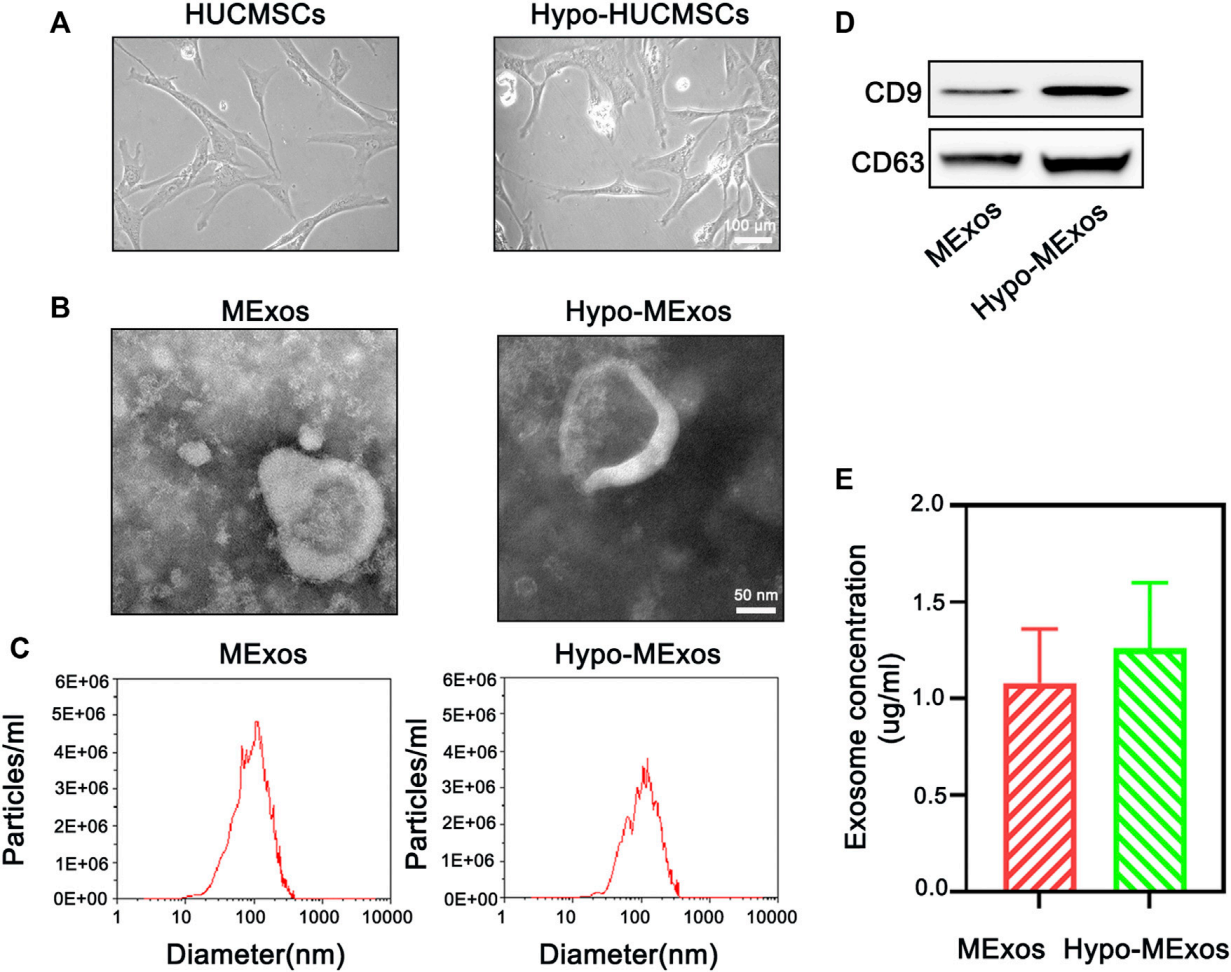
Characterization of HUCMSC-derived exosomes under normoxic conditions (MExos) and hypoxic conditions (Hypo-MExos). **(A)** Morphology of HUCMSCs and Hypo-HUCMSCs observed under a phase-contrast microscope. **(B)** TEM images of MExos and Hypo-MExos. **(C)** Particle size distributions of exosomes detected by NTA. **(D)** Western blot illustrating the characteristic surface markers of exosomes, CD9 and CD63. **(E)** Exosome concentration was determined by BCA quantification.

MExos and Hypo-MExos were isolated from the culture medium supernatant of normoxic and hypoxia-stimulated MSCs. Then, the free cell debris and large vesicles were removed by ultracentrifugation. The MExos and hypo-Mexos were characterized by NTA, TEM, and Western blotting. The macromorphology of the isolated MSCs was observed using a phase contrast optical microscope (Figure 1A), which showed no significant changes in either the HUCMSCs group or the Hypo-HUCMSCs group. The micromorphology of the extracted exosomes was observed by TEM, which showed typical cup-shaped and smooth double-layer structures (Figure 1B). The integrity of the original morphology of exosomes was maintained after hypoxic pretreatment. The NTA results showed that the peak particle sizes of MExos and Hypo-MExos were 113.1 and 129.1 nm, respectively, while the median diameters of both MExos and Hypo-MExos were 136.5 and 149.0 nm, respectively (Figure 1C). Western blotting results indicated that representative markers of exosomes, including CD9 and CD63, were expressed in the MExos and Hypo-MExos (Figure 1D). The exosome concentration of the Hypo-MExos group was quantified by bicinchoninic acid (BCA) protein assay, which showed an increasing trend but was not significantly different from that of the MExos group (Figure 1E). The results showed that after hypoxic pretreatment, the characterization of exosomes conformed to the identification standard of exosomes.

### Synthesis, characterization, and assessment of 3D-printed scaffolds

**FIGURE 2:**
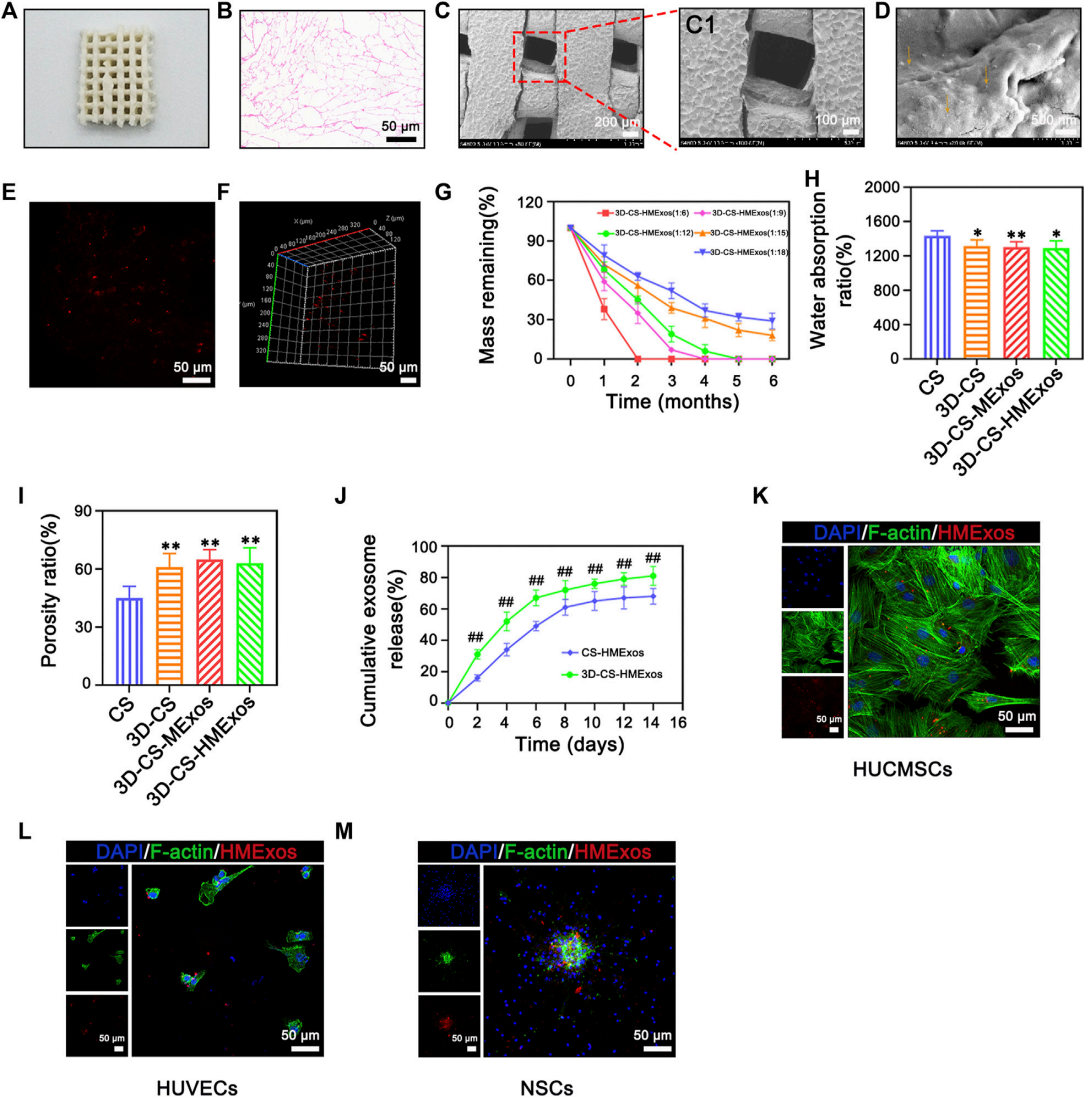
Characteristics of 3D-CS-HMExos. **(A)** General structure of 3D-CS-HMExos. **(B)** HE staining revealed the internal structure of 3D-CS-HMExos. **(C)** SEM micrographs of 3D-CS-HMExos. C1 is an enlarged view in the dashed box. **(D)** SEM image of 3D-CS-HMExos. The yellow arrow showed exosomes loaded on 3D-CS-HMExos. **(E)** Image of exosomes labelled with PKH26 in 3D-CS-HMExos. **(F)** 3D immunofluorescence staining images revealed the distribution of Exos in 3D-CS-HMExos **(G)** Degradation rate of 3D-CS-HMExos with different ratios of collagen and silk fibroin within 6 months. **(H)** Water absorption of scaffolds. **(I)** Porosity ratio analysis of the four groups. High porosity can increase the scaffold surface area/volume ratio, which is beneficial for exosome adhesion. **(J)** Cumulative release profile of exosomes from 3D-CS-HMExos and CS-HMExos. **(K–M)** Representative immunofluorescence staining images showed that the exosomes released from the scaffold could be phagocytized by HUCMSCs **(K)**, HUVECs **(L)** and NSCs **(M)** *in vitro*. Nuclei were stained with DAPI (blue), exosomes were stained with PKH26 (red), and the cytoskeleton was stained with F-actin (green). *p < 0.05, **p < 0.01 vs. CS. ^#^ *p* < 0.05, ^##^ *p* < 0.01 vs. CS-HMExos.

3D-CS-HMExos were synthesized by mixing Hypo-MExos into collagen/silk fibroin, wherein its porous structure supplies conditions for the adsorption and the sustained release of Hypo-MExos (Figure 2A). Its porous interconnected structures within the scaffolds suitable for cargo delivery were also observed with HE staining (Figure 2B). The porous structure inside 3D-CS-HMExos was displayed by SEM (Figures 2C,C1). The surface morphology of the scaffolds imaged a continuous and interconnected porous 3-dimensional network structure. SEM images also showed that Hypo-MExos can be firmly embedded and adsorbed on 3D-CS-HMExos (Figure 2D). The distribution of PKH 26-labelled Hypo-MExos on 3D-CS-HMExos was observed under CLSM (Figure 2E), and the 3D reconstruction image indicated the even distribution of PKH 26-labelled Hypo-MExos in 3D-CS-HMExos (Figure 2F). To develop an appropriate mixing ratio of the scaffolds, we selected collagen of different proportions with silk fibroin at 1:6, 1:9, 1:12, 1:15, and 1:18 mass ratios to detect the degradation ratio. The degradation ratio of scaffolds was measured at 1, 2, 3, 4, 5, and 6 months (Figure 2G). The ratio of the 1:12 group degraded 30% at 2 months and completely degraded at 5 months, which was suitable for the TBI repair process in this study. Therefore, the scaffold with 3D-CS-MExos and 3D-CS-HMExos with a mass ratio of 1:12 was used in the next experiment. The water absorption in PBS (pH 7.4) was shown in Figure 2H. These 3D-CS scaffolds exhibited a lower water absorption rate compared to CS (Figure 2H). The porosity of 3D-CS, 3D-CS-MExos, and 3D-CS-HMExos was significantly different from that of CS (Figure 2I). In addition, we immersed the fabricated CS-HMExos and 3D-CS-HMExos in PBS and detected exosomes released into PBS at different time points by BCA to calculate the release profile of exosomes (Figure 2J). HMExos loaded in the 3D-CS-HMExos exhibited continuous release for up to 14 days. The cellular uptake of hypo-MExos by cells was demonstrated in HUCMSCs, HUVECs and NSCs (Figures 2K–M). The results showed that PKH-26-labelled HMExos were scattered around the nuclei, indicating that exosomes can be normally phagocytized by HUCMSCs, HUVECs and NSCs at 1 day after incubation. In this study, 3D-CS-HMExos sustained the release of exosomes as a graft support, which further guaranteed their potential repair function in TBI.

### *In vitro* evaluation of the scaffolds

**FIGURE 3:**
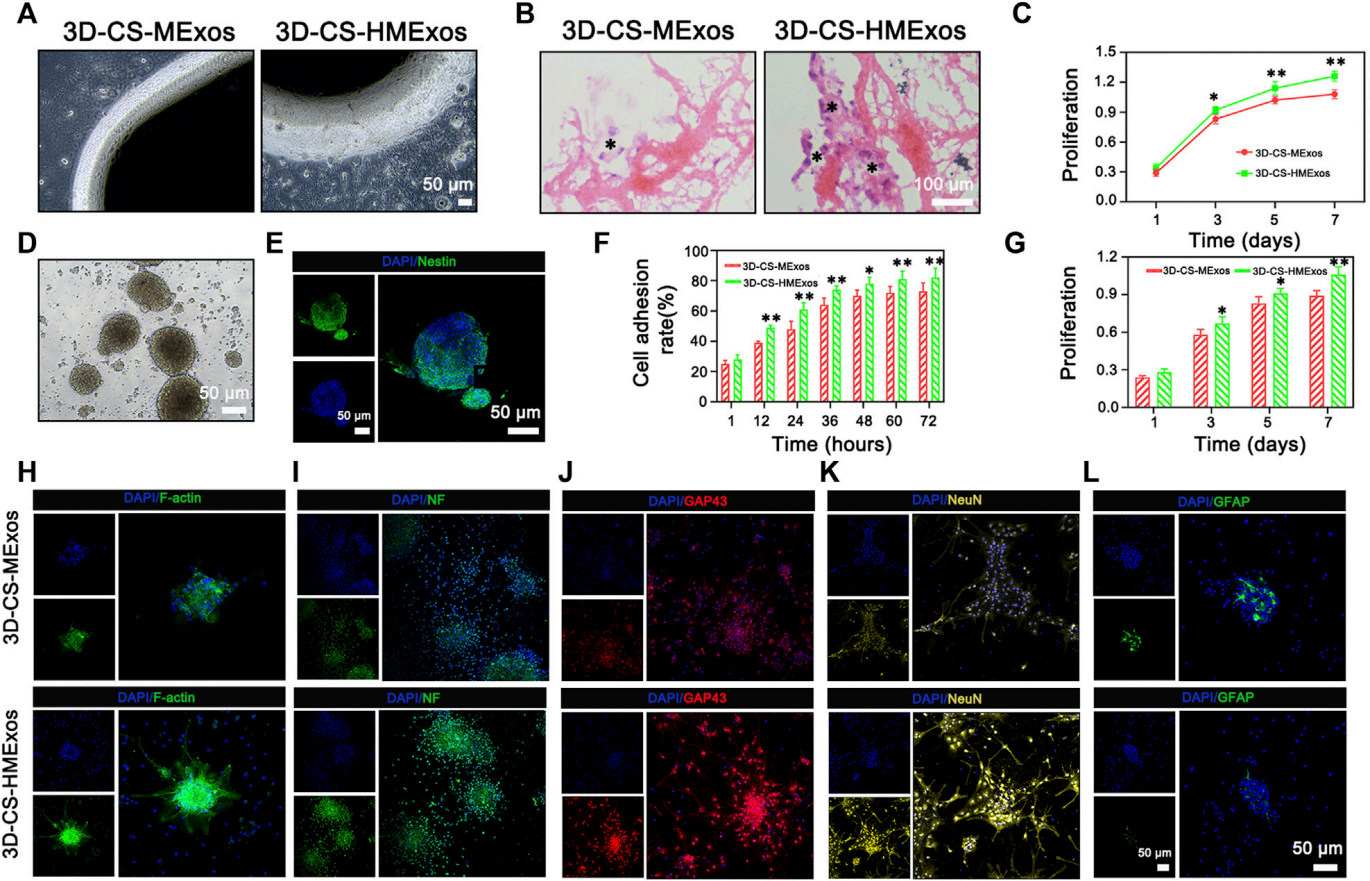
*In vitro* biological response of NSCs and HUCMSCs to 3D-CS-MExos or 3D-CS-HMExos. **(A)** Representative images of HUCMSCs coincubated with 3D-CS loaded with MExos or HMExos under a phase contrast microscope. **(B)** The morphology of 3D-CS-MExos and 3D-CS-HMExos after 7 days of coincubation with HUCMSCs assessed by HE staining. **(C)** Cell proliferation evaluated by CCK-8 assay after 1, 3, 5, and 7 days of culture in the scaffolds. **(D)** Representative images of NSCs under a phase contrast microscope after culture for 7 days. **(E)** Immunofluorescence staining identification verifies NSCs. Green and blue represent Nestin and DAPI, respectively. **(F)** Quantification analysis of the NSC adhesion rate of the 3D-CS-MExos/HMExos scaffold. **(H)** Cell adhesion analysis of NSCs was evaluated by cytoskeleton staining. Green: F-actin; blue: DAPI. **(G)** CCK-8 analysis of NSCs was also used to evaluate the biocompatibility of scaffolds *in vitro*. The proliferation rate of NSCs on the 3D-CS-HMExos scaffold was significantly higher than that on the 3D-CS-MExos scaffold. **(I–L)** Immunofluorescence staining analysis to verify the differentiation of NSCs after 7 days of incubation in scaffolds with NF, GAP43, NeuN, and GFAP antibodies. *p < 0.05, **p < 0.01 vs. 3D-CS-MExos.

*In vitro* assessments were applicable to evaluate the cell compatibility of scaffolds and to detect whether the scaffolds offered a conducive microenvironment for NSC differentiation. To analyse the biocompatibility and regenerative capability in response to exosomes, 3D-CS-MExos and 3D-CS-HMExos were incubated with HUCMSCs and NSCs, respectively. Subsequently, the specimens were imaged using a phase contrast microscope (Figure 3A). The morphology images showed no significant changes in the coincubation of 3D-CS-MExos or 3D-CS-HMExos with HUCMSCs. Additionally, HE staining showed excellent biocompatibility of 3D-CS-HMExos cocultured with HUCMSCs, with relatively large amounts of cell proliferation and deposition in the 3C-CS-HMExos group (Figure 3B). The proliferation of HUCMSCs coincubated in 3D-CS-MExos or 3D-CS-HMExos was demonstrated by CCK8 assay (Figure 3C). The proliferation increased slightly on the 3D-CS-MExos scaffold but rapidly increased on the 3D-CS-HMExos scaffold.

To further explore the effect of the HMExos on NSCs, NSCs were isolated (Figure 3D) and identified using immunofluorescence staining (Figure 3E). The results showed that the protein expression of the surface marker of NSCs, nestin, was abundantly expressed. Since the cellular morphology and behavior of NSCs depend partly on the proper organization of actin filaments, we performed cellular assays to examine cell adhesion and morphology, which are critical to assessing exosome function in the adhesion, proliferation, and differentiation of NSCs. As expected, the cell adhesion ability of the NSCs on 3D-CS-HMExos was significantly increased compared with that on 3D-CS-MExos (Figures 3F,H), indicating that hypoxia-induced exosomes might play important roles in mediating NSC function. The proliferation of NSCs cocultured with 3D-CS-MExos or 3D-CS-HMExos scaffolds is shown in Figure 3G. NSCs were observed to significantly proliferate in both 3D-CS scaffolds incubated with MExos and HMExos within 7 days of culture. The numbers of NSCs in the 3D-CS-HMExos scaffold were higher than those in the 3D-CS-MExos scaffold at 3, 5, and 7 days (Figure 3G). The NSC viability at 3, 5, and 7 days was consistent with the results obtained for HUCMSCs. In our study, we verified markers of neural differentiation after 7 days of culture with immunofluorescence staining staining analysis, focusing on differentiated axons, nerve fibers, neurons, and astrocytes. Among the protein expression levels, NF, GAP43, NeuN, and GFAP indicated mature differentiated nerve fibers, axonal neurons, and astrocytes, respectively (Figures 3I–L). The immunofluorescence staining results showed that the NSCs cocultured with the 3D-CS-HMExos scaffold expressed relatively high mature neuronal differentiation intensity and axonal proteins compared with those cocultured with the 3D-CS-MExos scaffold. However, the exact opposite result of astrocyte expression was observed in the two groups. Taken together, these findings support the ability of the 3D-CS-HMExos scaffold to enhance the neural differentiation potential of NSCs.

### Neurological function evaluation of MExos-loaded 3D-printed collagen/silk fibroin scaffolds and Hypo-MExos-loaded 3D-printed collagen/silk fibroin scaffolds implants in traumatic brain injury beagle dogs

**FIGURE 4:**
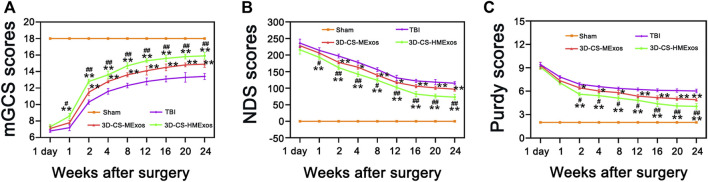
Hypoxia-induced exosomes combined with 3D-printed collagen and silk fibroin scaffolds improved locomotor function after TBI. mGCS scores **(A)**, NDS scores **(B)**, and purdy scores **(C)** were evaluated and analysed at 1 day and 1, 2, 4, 8, 12, 16, 20, and 24 weeks after TBI. *p < 0.05, **p < 0.01 vs. TBI group. ^#^ *p* < 0.05, ^##^ *p* < 0.01 vs. 3D-CS-MExos group, *n* = 5.

The efficiency of 3D-CS-MExos and 3D-CS-HMExos in promoting locomotor function repair was further evaluated using the TBI beagle dog model. The mGCS, NDS and Purdy scores were recorded and quantitated to evaluate the recovery of motor function in TBI dogs implanted with 3D-CS-MExos and 3D-CS-HMExos (Figures 4A–C). Each group exhibited a similar neurological impairment on the first day after TBI. Consistent with the NDS scores, the Purdy scores suggested tremendous behavioral function recovery in the treatment group compared to the TBI group, which was also observed in the mGCS scores after implantation of 3D-CS-MExos and 3D-CS-HMExos for 7 days. As shown in Figure 4, significant differences in mGCS, NDS and Purdy scores were detected in the 3D-CS-HMExos group compared to the TBI group and 3D-CS-MExos group at each time point after 2 weeks. Generally, the TBI beagles implanted with 3D-CS-HMExos revealed the best locomotor functional recovery. This finding suggests that 3D-CS-HMExos could further promote the locomotor functional recovery of beagles with TBI to a certain extent.

### Regeneration of nerve fibers, myelin sheath, and axon regeneration after hypoxia-induced human umbilical cord mesenchymal stem cells-exosomes loaded 3D-printed scaffolds implantation

**FIGURE 5:**
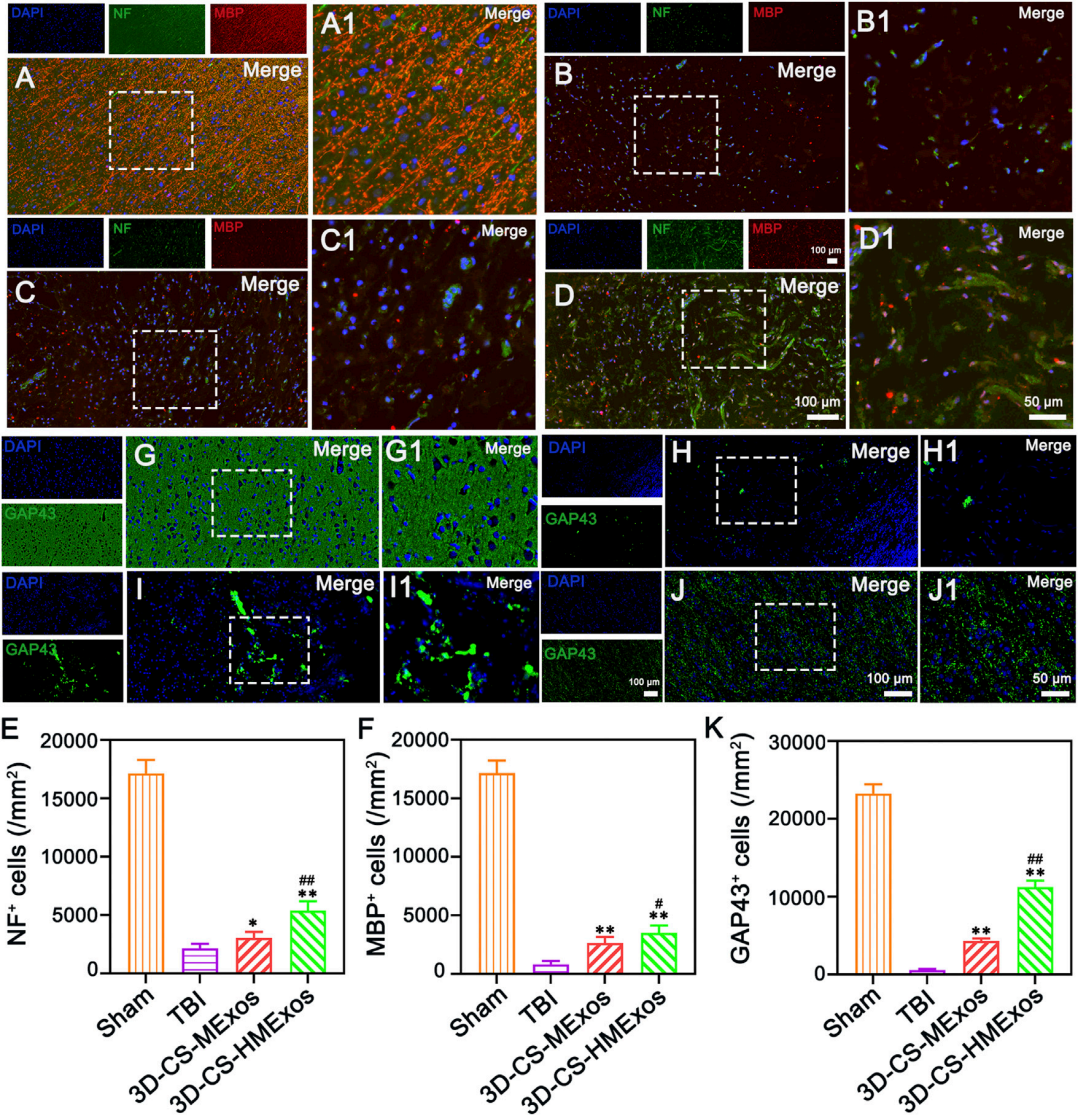
Nerve fiber regeneration, myelination, and axonal outgrowth after TBI. **(A–D)** 6 months after TBI, the NF, and MBP staining results identified regenerated nerve fibers that had undergone myelination. **(E,F)** Quantification of the relative density of NF- and MBP-positive cells in each group. **(G–J)** Axonal regeneration (GAP43^+^) was detected in lesion sites of canines treated with a hypoxia-modified exosome scaffold (3D-CS-HMExos). **(K)** Quantification of newborn axons in the lesion area of dogs among each group. The amplification views of the injured sites, in which the signals of NF, MBP, and GAP43 in regions between the black dotted bordered rectangle are presented in (A1), (B1), (C1), and (D1), respectively. The image on the right is an enlarged image of the image in the white box of the image on the left. *p < 0.05, **p < 0.01 vs. TBI group. ^#^ *p* < 0.05, ^##^ *p* < 0.01 vs. 3D-CS-MExos group, *n* = 5.

To verify nerve fiber regeneration, myelination, axonal outgrowth and after TBI, NF, MBP, and GAP43 were further investigated by immunofluorescence staining. Nerve fiber regeneration was additionally evaluated with the nerve fibers marker neurofilaments (NF). Along with axonal extension, newly generated myelination emerged at the periphery of regenerated axons (Figures 5A–D,F). As shown, immunofluorescence staining of NF and MBP indicated significant nerve fiber regeneration and remyelination associated with the 3D-CS-HMExos scaffold implanted at the brain lesion site (Figures 5A–F). There were more GAP43-positive cells at the lesion site in the 3D-CS-HMExos group than in the TBI and 3D-CS-MExos groups (Figures 5G–K). These results suggest that implantation of hypoxia-induced exosomes associated with 3D-CS scaffolds could provide a suitable microenvironment for neuroregeneration and further enhance axon formation and myelination at the injury site.

### Establishment of synaptic connections after hypoxia-induced human umbilical cord mesenchymal stem cells-exosomes loaded 3D-printed collagen/silk fibroin scaffold implantation

**FIGURE 6:**
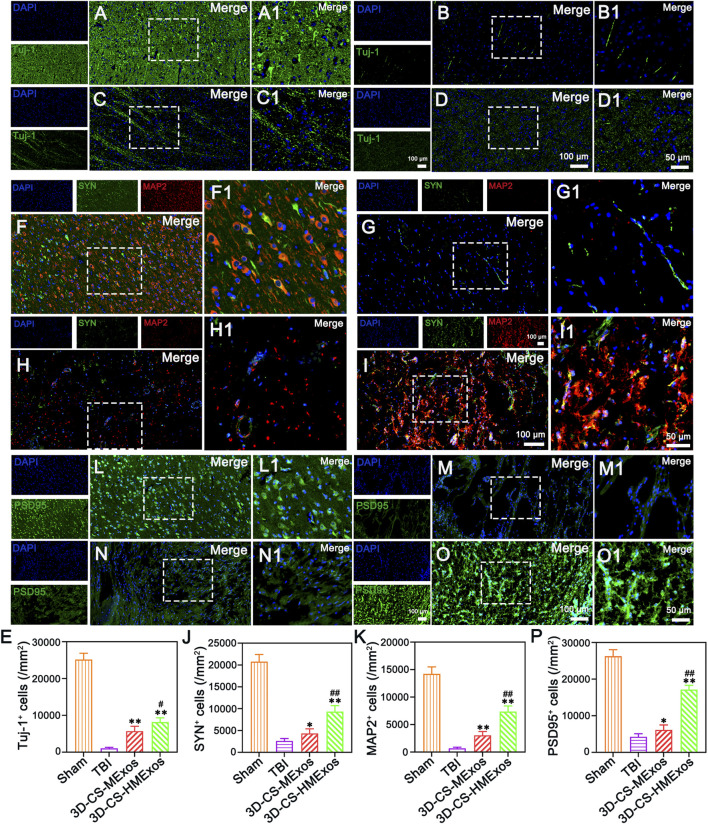
Regenerated neurons and synaptic connection formation at 6 months after TBI. **(A–D)** Representative images of Tuj-1-positive neurons in the lesion site of beagles in each group. **(E)** Quantitative analysis of Tuj-1 in injured brain tissues in the Sham, TBI, 3D-CS-MExos, and 3D-CS-HMExos groups. **(F–I)** Double-labelling of SYN and MAP2 immunofluorescence staining staining images of synapse formation at the lesion site, green: SYN; red: MAP-2. **(J,K)** SYN- and MAP-2-positive cells in the four groups at 6 months after the operation. **(L–O)** Representative immunofluorescence staining images showed another hallmark protein of synapses, PSD95, which serves as an important surface marker in neuronal excitatory postsynaptic membranes. **(P)** Quantitative analysis of PSD95-positive signals in the four groups, indicating that hypoxia-pretreated HUCMSCs-exosomes combined with 3D-printed collagen/fibroin scaffolds could further promote the formation of synaptic connections after TBI. The image on the right is an enlarged image of the image in the white box of the image on the left. *p < 0.05, **p < 0.01 vs. TBI group. ^#^ *p* < 0.05, ^##^ *p* < 0.01 vs. 3D-CS-MExos group, *n* = 5.

The neuronal marker Tuj-1 was used to assess the condition of neurons in the injured area after TBI. Implantation of 3D-CS-HMExos further increased the number of Tuj-1 positive cells in the injured area after TBI compared with implantation of 3D-CS-MExos, indicating that hypoxia-induced HUCMSCs-exosomes are beneficial for increasing the number of neurons in the injured area after TBI (Figures 6A–E). Synaptic connection formation was further investigated by immunofluorescence staining staining of the synaptic connection markers synaptophysin (SYN) and postsynaptic density protein-95 (PSD95) and microtubule associated protein 2 (MAP2), which represent synaptic reconstruction and reflect the compensatory regeneration of nerve fibers, myelin sheaths, and axons. As the results showed, immunofluorescence staining of SYN, PSD95, and MAP2 in the lesion area revealed the superior synaptic regenerative capacity of HUCMSCs-exosomes combined with the 3D-CS scaffold (Figures 6F–P). Notwithstanding, the therapeutic effect of the 3D-CS-HMExos scaffold was significantly better than that of the 3D-CS-MExos scaffold (Figures 6F–P). In particular, the images illustrated that tighter synaptic connections were formed in the 3D-CS-HMExos group in the injury area than in the TBI and 3D-CS-MExos groups (Figures 6F–P). The evaluation showed consistent results with locomotor function recovery trends for subsequent assessments, demonstrating the effective neuroregenerative capacity of the 3D-CS-HMExos scaffold.

### Proangiogenic functions of hypoxia-stimulated human umbilical cord mesenchymal stem cells-exosomes *in vivo*

**FIGURE 7:**
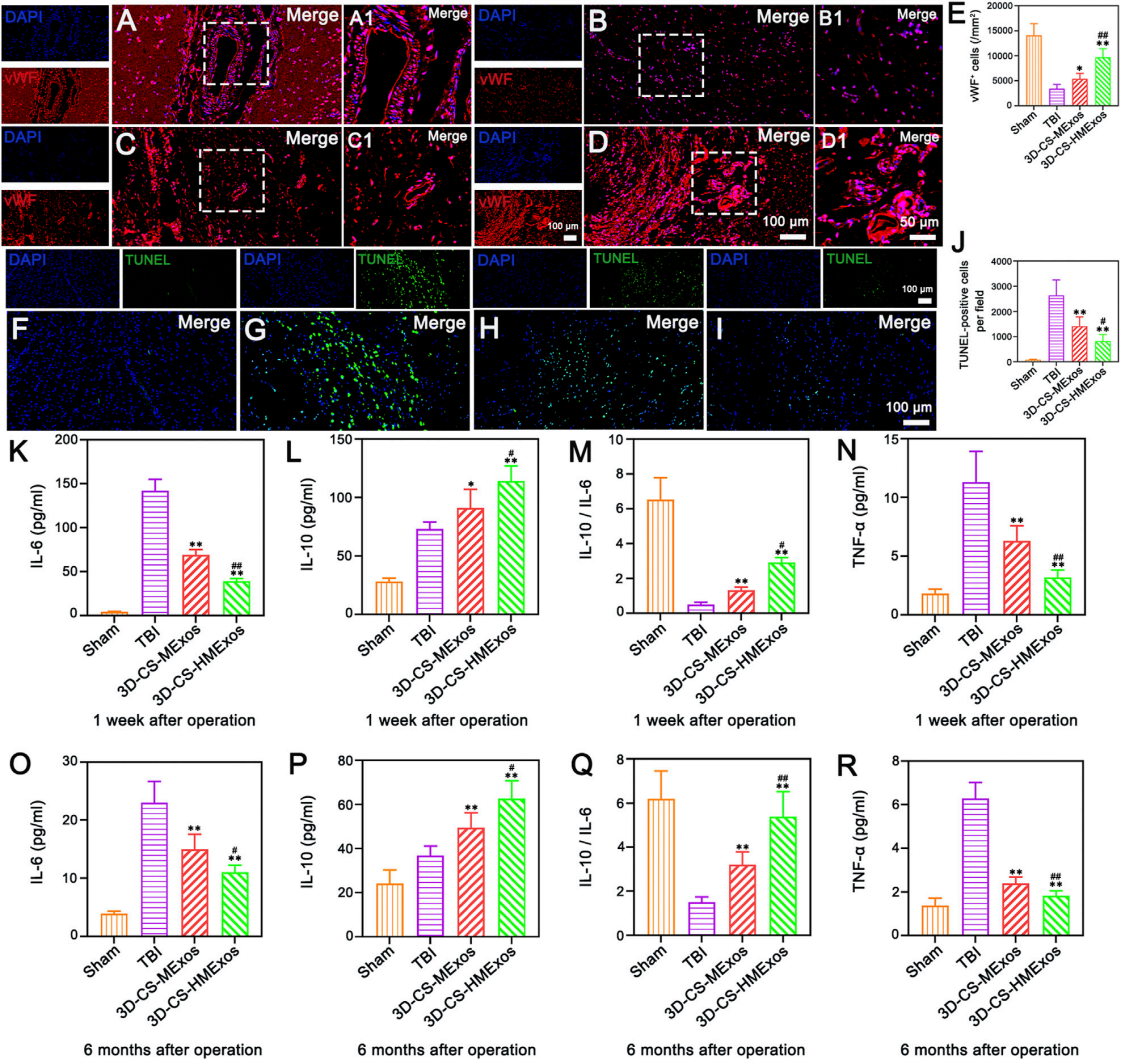
Hypoxia-induced exosomes combined with 3D-printed collagen and silk fibroin scaffolds significantly enhanced angiogenesis, inhibited apoptosis, and alleviated inflammation after TBI. **(A–D)** Representative image of vWF immunofluorescence staining (red) in the lesion area. **(A–D)** show the full view of the injured area, with the boxed adjacent and lesion areas magnified in A1, B1, C1 and D1, respectively. Nuclei were stained with DAPI (blue). **(E)** Quantitative analysis of vWF-positive cells in the lesion area in the four groups. **(F–I)** Representative images revealing TUNEL-positive cells (green) indicative of cell apoptosis within the injured area at 6 months after TBI. Note that the 3D-CS-HMExos group showed fewer apoptotic cells than the TBI and 3D-CS-MExos groups at 6 months after TBI. **(J)** Quantitative analysis of TUNEL-positive cells. **(K–R)** Graphs showing the expression of the inflammatory factors IL-6 **(K,O)**, IL-10 **(L,P)**, IL-10/IL-6 **(M,Q)**, and TGF-α **(N,R)** in the acute (1 week) and chronic phases (6 months) after the operation. Note that lower levels of IL-6 and TGF-α and higher levels of IL-10 were observed in the 3D-CS-HMExos group than in the 3D-CS-MExos group at 1 week and 6 months after the operation. In general, 3D-CS-HMExos diminished proinflammatory cytokine (IL-6, TGF-α) release but enhanced anti-inflammatory cytokine (IL-10) release after TBI. The image on the right is an enlarged image of the image in the white box of the image on the left. *p < 0.05, **p < 0.01 vs. TBI group. ^#^ *p* < 0.05, ^##^ *p* < 0.01 vs. 3D-CS-MExos group, *n* = 5.

Vascularization effects were further evaluated to investigate the alleviation of the microenvironment after TBI by hypoxia-induced HUCMSCs-exosomes. The distribution of von Willebrand factor (vWF)-positive staining was present in the damaged brain tissue of each group (Figures 7A–D). In comparison, the 3D-CS-HMExos group exhibited a larger amount of blood vessel structures. The vWF-positive structures were quantified and shown to be closely associated with 3D-CS-HMExos engraftment (Figure 7E). In contrast to the TBI and 3D-CS-MExos groups, the 3D-CS-HMExos group significantly further increased vWF expression at the center of the lesion, implying that HMExos regulated angiogenesis at the implantation site.

### The effect of hypo-MExos-loaded 3D-printed collagen/silk fibroin scaffolds on apoptosis after traumatic brain injury

To further investigate whether the implantation of 3D-CS-HMExos could ameliorate neuron survival, TUNEL staining was performed at 6 months after TBI. Fewer TUNEL-positive cells were observed in the 3D-CS-HMExos group than in the TBI group and the 3D-CS-MExos group (Figures 7F–J). These findings demonstrated that the 3D-CS-HMExos scaffold has the best utility of significantly inhibiting apoptosis after TBI. Since plasma inflammatory factors are the primary mediators of inflammation after injury, we studied the expression of TNF-a, IL-6, and IL-10 at 1 week and 6 months in peripheral venous blood plasma to confirm whether the 3D-CS-HMExos scaffold can alter the expression of these plasma inflammatory factors (Figures 7K–R). Consistent with the results of the analysis at 1 week, the expression of IL-6 and TNF-a was lower in the 3D-CS-HMExos group than in the TBI and 3D-CS-MExos groups, while the expression of IL-10 in the 3D-CS-HMExos group was higher at 6 months after the operation (Figures 7K–R). Together, these results indicate that the implantation of 3D-CS-HMExos could further inhibit apoptosis and reduce inflammation after TBI.

## Discussion

Some previous studies have only assessed the influence of 3D-CS implants on regeneration after TBI. The effect of exosomes with or without hypoxia combined with 3D-CS scaffolds on large animals remains unclear. Therefore, in the current study, the influence of hypoxia-induced HUCMSCs-exosomes (HMExos) combined with 3D-CS (3D-CS-HMExos) scaffolds on post-TBI regenerative ability was explored. In this study, a TBI model of beagles was established by a modified electrically controlled cortical impactor approach. A 3D printing technique was used to fabricate scaffolds to pad the brain cavity of TBI beagles. The biocompatibility of 3D-CS-HMExos loaded with HMExos was verified by coincubation with HUCMSCs and NSCs, the most commonly employed cell sources for brain tissue engineering. The results of our experiments confirmed that 3D-CS-HMExos could serve as a novel strategy based on exosomes and biomaterials for brain tissue regeneration.

A defect cavity forming *in situ* after TBI is one of the devastating problems restricting its nerve repair. Current tissue engineering repair strategies are altering to try to raise this challenge, with innovative strategies from simple scaffold implantation to scaffold-carrying stem cell or growth factor delivery. Natural biomaterials were ideal scaffolding materials to fill brain defect cavities and supported the ingrowth and integration of cells (Liu Y. et al., 2020). For example, the utility of chitosan derived from chitin has been demonstrated to have beneficial effects on enhanced regeneration in rats, holding safety *in vivo* applications (Mo et al., 2010; Skop et al., 2014).

Multiple studies have been implemented to demonstrate the effect of 3D printing scaffolds for neuroregeneration. Therefore, there is an urgent need to develop composite natural biomaterials that can better repair brain injury. As one of the constituents of the extracellular matrix, collagen has several attractive features, such as biodegradability, biocompatibility, and low immunogenicity for neural repair (Zhang L. et al., 2021), but it still has many shortcomings that need to be further improved to afford a beneficial microenvironment for TBI repair. Silk fibroin, a biomaterial obtained from silkworm cocoons, has been widely investigated due to its favorable biocompatibility. Nevertheless, some defects of silk fibroin, such as frangibility and high solubility, restrict its application (Li et al., 2018). To adapt to different repair needs and overcome their own limitations, blending silk fibroin with other biomaterials has attracted increasing attention. In the current study, as a biodegradable mixture, the collagen/silk fibroin composite overcame the defects of pure collagen and silk fibroin and showed remarkable biocompatibility, appropriate biodegradability, and mechanical properties. Therefore, it could be a promising tissue engineering material for TBI. In this study, 3D-CS-HMExos implants were developed with the incorporation of exosomes into scaffolds. One of the benefits of 3D-CS-HMExos was their inherent porous structure, consisting of pore size, network connection and size distribution, which have been shown to have significant effects on tissue regeneration (Duan et al., 2016; Bae et al., 2021). Another advantage was its biocompatibility and biodegradability, ensuring that scaffolds induce minimal inflammatory responses after implantation *in vivo*. Compared with similar articles related to collagen/fibroin scaffolds. The scaffolds in our study prepared by 3D printing technology have more uniform internal pore size and are more suitable for cytokine intercommunication than scaffolds prepared by simple mixing of collagen and silk fibroin alone. Moreover, collagen/silk fibroin scaffolds equipped with natural exosomes have better biocompatibility and repair effect than scaffolds reported in other literatures.

As important mediators of cell‒cell communication, exosomes can regulate immunity, which plays a key role in neuroregeneration. Numerous studies have reported the effect of exosome therapy on the repair of TBI. The exosomes derived from MSCs promote TBI repair by regulating paracrine mechanisms. The exosomes derived from MSCs carry soluble factors, nucleic acids, lipids, and proteins that may enhance neural regeneration and thus improve neurological recovery. Alterations in the microenvironment usually influence the cargo packaging of exosomes and may shift the therapeutic functions of exosomes. Oxygen concentration was an important factor affecting the cell biology of MSCs (Mohyeldin et al., 2010), and hypoxia stimulation makes MSCs in an activated/stressed state, which could secrete a large number of exosomes (Gonzalez-King et al., 2017). The measurement of hypoxia handling for the production of enhanced exosomes is critical in this strategy, which can trigger angiogenesis and enhance cell tolerance to hypoxia. In this study, hypoxia-induced exosomes derived from HUCMSCs loaded 3D-CS-HMExos were applied to explore the therapeutic effect on nerve regeneration and motor function recovery after TBI.

Sufficient exchange of trophic factors and metabolites in biomaterials facilitates the survival and regeneration of surrounding nerve cells. For that, a porous structure is required for the 3D-printed scaffold. The results of HE staining and SEM proved that 3D-CS-HMExos presented a porous network structure and that the exosomes were bound to the inner surface of 3D-CS-HMExos. The above results verified that exosomes could be successfully loaded onto 3D-CS-HMExos. Additionally, the exosomes loaded on 3D-CS-HMExos could be released continuously by more than 80% in 14 days, ensuring an uninterrupted repair of nerve damage.

The degradation rate of transplanted carriers affects the efficacy of delivered exosomes. Moreover, the suitable degradation rate is crucial for the integration of transplanted carriers into the host tissue. To select the best degradation profile of the 3D-CS-HMExos scaffold, printed constructs for different ratios of collagen and silk fibroin (1:6, 1:9, 1:12, 1:15, and 1:18) were incubated in PBS at 37°C, having similar ionic and temperature conditions to *in vivo* conditions. The results showed that the scaffold was completely degraded at 5 months at a ratio of 1:12 collagen to silk fibroin, which best matched the time point set in this study.

Cell proliferation and adhesion were important to determine the biological properties of grafted biomaterials for neuronal regeneration (Unal et al., 2019). We performed cytoskeleton staining and metabolite analysis with CCK-8 at specific time points to verify the cytotoxicity on each scaffold. Cytoskeleton staining images showed the encapsulated cell proliferation of HUCMSCs or NSCs within 7 days. However, proliferation increased slightly on 3D-CS-MExos but rapidly increased on 3D-CS-HMExos. Cytoskeleton imaging revealed that the NSCs in each group had a better range of expansion area, further indicating that the 3D-CS-HMExos had beneficial adhesion for NSCs. Cell differentiation is essential for posttraumatic nerve repair. Therefore, we examined the effect of 3D-CS-HMExos scaffolds on the differentiation of NSCs *in vitro*. During morphology imaging of immunofluorescence staining on each scaffold, the cells on the 3D-CS-HMExos groups at 7 days after coculture showed the greatest number of nerve fibers, axons and neurons and the lowest number of astrocytes compared to the 3D-CS-MExos group on the same date. NSCs can highly proliferate during the self-renewal period, whereas the proliferation rate rapidly decreases during neuronal differentiation (Homem et al., 2015). This result may shed light on the role of hypoxia-induced exosome loading on 3D-CS scaffolds in regulating the proliferation of NSCs and differentiation into neurons.

Insufficient vessel growth associated with injury remains an unresolved issue, restricting neuroregeneration after TBI. The hypoxic microenvironment has been reported to upregulate the expression of proangiogenic factors in MSCs and enhance their wound-healing ability (Tong et al., 2016; Mu et al., 2022). According to reports, exosomes derived from hypoxia-induced MSCs have proven to be effective in treating femoral head osteonecrosis by promoting angiogenesis (Yuan et al., 2021). In addition, studies have shown that exosomes from hypoxia-preconditioned human umbilical vein endothelial cells (HUVECs) promote MSC angiogenic function after spinal cord injury (Li et al., 2022). The present work attempted to investigate whether exosomes derived from HUCMSCs upon hypoxia stimulation could mediate angiogenesis. The results showed that hypoxia-induced exosomes promoted angiogenesis *in vivo*, particularly at 6 months after TBI. The immunofluorescence staining results of vWF, an angiogenesis marker, were significantly higher on 3D-CS-HMExos than on 3D-CS-MExos. These findings provide new insights into the exosome-mediated promotion of neurodegeneration and the surface design of biomaterials from the perspective of angiogenesis.

The ultimate goal of hypoxia-induced exosomes loaded 3D-CS-HMExos delivery was to enhance the neuroregeneration of the damaged brain. We next investigated the *in vivo* behavior of grafted HMExos within 3D-CS-HMExos in TBI beagle models by directly implanting 3D-CS-HMExos into the injury area of the beagles brain. In this study, we verified markers of nerve fibers at 6 months after implantation with immunofluorescence staining analysis, focusing on the regeneration of functional nerve fibers, axon, myelination, regeneration of neurons and the establishment of synaptic connections under higher magnification. Immunofluorescence staining quantitative analysis showed that the protein expression of the nerve fibers, myelination, axons, neurons, and synapse formation markers NF/MBP, GAP43, Tuj-1, SYN/MAP2, and PSD95 was higher on 3D-CS-HMExos than on 3D-CS-MExos.

We generally recognize that apoptosis at the injury site is closely associated with neurological function impairments. Hence, we analysed apoptosis using TUNEL staining. The results indicated that 3D-CS-HMExos significantly inhibited apoptosis after TBI. Neuroinflammation was a key component of the pathological environment during TBI pathogenesis (Johnson et al., 2013). In response to TBI, a large number of proinflammatory and anti-inflammatory cytokines were released (Loane and Kumar, 2016). The balance between inflammation and antiinflammation could be measured by the levels and ratios of IL-6 and IL-10 (Jiang et al., 2021). In our study, we found that 3D-CS-HMExos implants could lessen neuroinflammation, manifested by downregulating the expression of the proinflammatory factors IL-6 and TNF-α and increasing the level of the anti-inflammatory factor IL-10.

To verify the feasibility of using 3D-CS-HMExos in terms of motor function recovery, mGCS scores, NDS scores, and Purdy scores were used. The results indicated that implantation of 3D-CS-HMExos facilitated recovery of motor function after TBI, which might be related to regeneration of nerve fibers, axons and neurons, remyelination, and synaptic connection establishment at the injury site after TBI.

## Conclusion

In summary, our approach demonstrated that hypoxia-induced exosome carriers with a 3D-printed collagen/silk fibroin scaffold promoted neuroregeneration and motor function recovery after TBI. Moreover, the method allowed suppression of prolonged neuroinflammation and inhibition of nerve cell apoptosis after TBI. The combined use of the 3D-CS scaffold and hypoxia-induced exosomes increased the effectiveness of implantation after TBI by supporting angiogenesis and nerve regeneration at the lesion while suppressing chronic neuroinflammation and nerve cell apoptosis. This study provides a novel treatment for TBI based on exosome-based therapy.

## Data Availability

The original contributions presented in the study are included in the article/supplementary material, further inquiries can be directed to the corresponding authors.
